# Emerging Applications of Deep Learning in Bone Tumors: Current Advances and Challenges

**DOI:** 10.3389/fonc.2022.908873

**Published:** 2022-07-19

**Authors:** Xiaowen Zhou, Hua Wang, Chengyao Feng, Ruilin Xu, Yu He, Lan Li, Chao Tu

**Affiliations:** ^1^ Department of Orthopaedics, The Second Xiangya Hospital, Central South University, Changsha, China; ^2^ Xiangya School of Medicine, Central South University, Changsha, China; ^3^ Hunan Key Laboratory of Tumor Models and Individualized Medicine, The Second Xiangya Hospital, Central South University, Changsha, China; ^4^ Department of Radiology, The Second Xiangya Hospital, Central South University, Changsha, China; ^5^ Department of Pathology, The Second Xiangya Hospital, Central South University, Changsha, China

**Keywords:** bone tumor, sarcoma, deep learning, artificial intelligence, cnn, convolutional neural network

## Abstract

Deep learning is a subfield of state-of-the-art artificial intelligence (AI) technology, and multiple deep learning-based AI models have been applied to musculoskeletal diseases. Deep learning has shown the capability to assist clinical diagnosis and prognosis prediction in a spectrum of musculoskeletal disorders, including fracture detection, cartilage and spinal lesions identification, and osteoarthritis severity assessment. Meanwhile, deep learning has also been extensively explored in diverse tumors such as prostate, breast, and lung cancers. Recently, the application of deep learning emerges in bone tumors. A growing number of deep learning models have demonstrated good performance in detection, segmentation, classification, volume calculation, grading, and assessment of tumor necrosis rate in primary and metastatic bone tumors based on both radiological (such as X-ray, CT, MRI, SPECT) and pathological images, implicating a potential for diagnosis assistance and prognosis prediction of deep learning in bone tumors. In this review, we first summarized the workflows of deep learning methods in medical images and the current applications of deep learning-based AI for diagnosis and prognosis prediction in bone tumors. Moreover, the current challenges in the implementation of the deep learning method and future perspectives in this field were extensively discussed.

## 1 Introduction

Bone tumors occur in the musculoskeletal system and can be divided into primary and metastatic bone tumors. It is reported that the incidence of primary bone tumors is 2–3 per 100,000 people, accounting for about 6.2% of all tumors (1–4). Primary bone tumors can also be subdivided into benign, intermediate, and malignant tumors. Among them, osteosarcoma is the most prevalent primary malignant bone tumor and contributes to the second cause of death in adolescents and children (1, 5–8). Notably, without timely diagnosis and treatment, patients with a malignant bone tumor may suffer the risk of a worse prognosis, such as amputation or metastasis (2, 9, 10). Therefore, early detection and treatment are pivotal for limb salvage and reducing morbidity and mortality. Currently, the conventional diagnosis procedure for a bone tumor is a combination of clinical characteristics, imaging, and pathological examinations since bone tumors are rare. However, it is not easy for clinicians to achieve accurate and timely diagnoses since the available diagnostic process can be laborious, high cost, time consuming, and could be biased by clinicians’ expertise and experience (11, 12). Accordingly, there is an urgent need for a more rapid, reliable, and accurate diagnosis of bone tumors in clinical practice.

In the field of healthcare, artificial intelligence (AI) has a wide range of applications, including imaging and diagnostics, lifestyle management and supervision, nursing, emergency and hospital management, drug mining, virtual assistants, wearables, and more (13, 14). AI in the healthcare industry can tremendously improve the efficiency of clinical work and reduce the shortage of medical resources. Since deep learning technology began to be applied to image recognition tasks in 2012, their recognition performance has reached a high point in recent years. The research methods have been widely used in medical image analysis and processing tasks. Also, the applications of the deep learning method in medical images provide assistance for disease diagnosis automatically with shortened time, enhanced efficacy, and favorable accuracy (15).

Due to the advancement in AI, techniques based on deep learning such as segmentation, detection, classification, and enhancement have been successfully applied to the field of medical imaging (15–26), bringing new opportunities for building computer-aided medical imaging diagnosis system. In recent years, deep learning methods have been successfully used in musculoskeletal imaging for lesion identification and severity assessment, such as a fracture (27–36), knee lesion (37–42), osteoarthritis (43–47), and spinal degenerative lesion (48, 49). In addition, some models based on deep learning are adopted to assess bone age (50–55) and determine sex (56) on radiographs. Therefore, it is plausible to use deep learning methods to establish diagnostic models for bone tumors as well, which may greatly reduce the misdiagnosis and missed diagnosis rates of bone tumors. Recently, a growing number of studies have reported that deep learning-based AI models in bone tumor identification, classification, segmentation, and visual interpretation could improve diagnostic, prognostic, and predictive accuracy, demonstrating its great potential application in bone tumors. Moreover, often compared with deep learning, radiomics is also an advanced technology that is often cooperated with artificial intelligence, designed to extract, and analyze numerical radiological patterns based on quantitative image features, including geometry, size, texture, and intensity. It is well known that radiomics could be applied in disease prediction, prognosis, and monitoring (57, 58). The purpose of this review is to briefly describe the concepts and workflows of deep learning-based AI, extensively summarizing its up-to-date applications in bone tumors, as well as discuss the barriers to deep learning implementation and future directions in this field.

## 2 Artificial intelligence based on deep learning

AI can refer to a branch of computer science that can simulate human intelligence. AI is implemented in machines to perform tasks that require human knowledge in an automated manner (59). On the other hand, machine learning (ML) is a subset of AI and is defined as mathematical algorithms that enable a machine to make choices independently without any external human influence (60). Furthermore, deep learning, as a novel approach, is a subset of ML, but there are differences between ML and deep learning in data dependencies, hardware dependencies, feature engineering, problem-solving approach, execution time, and interpretability (61). Deep learning can learn to perform much more complex classification tasks from input such as images, text, or sounds, achieving superior performance to traditional machine learning (62). Accordingly, deep learning models contain many layers to build neural network architectures and need to be trained by a large set of labeled data.

**Figure 1:**
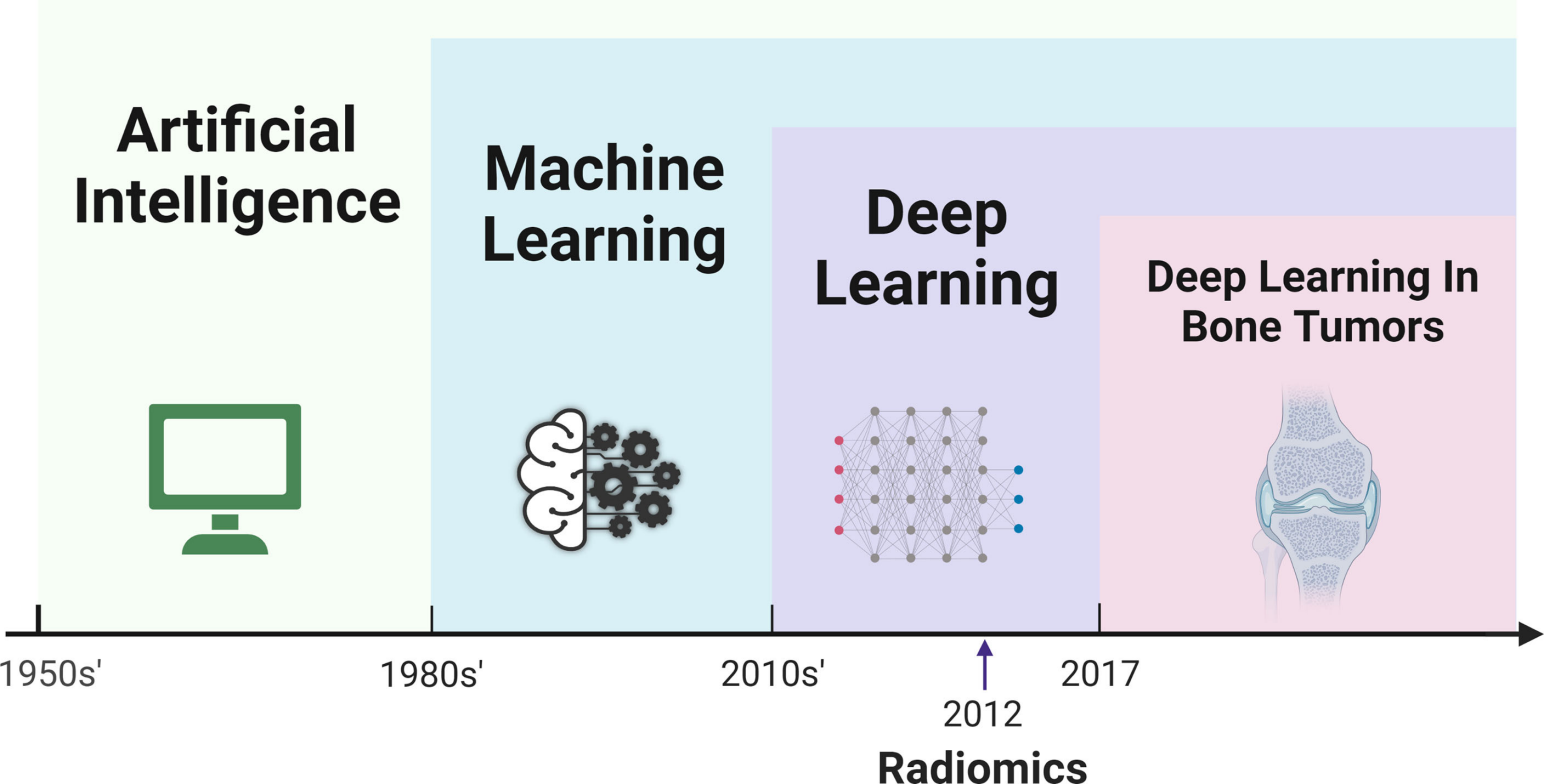
Timeline of the development of artificial intelligence, machine learning, radiomics, deep learning, and application of deep learning in field of bone tumor.

**Figure 2:**
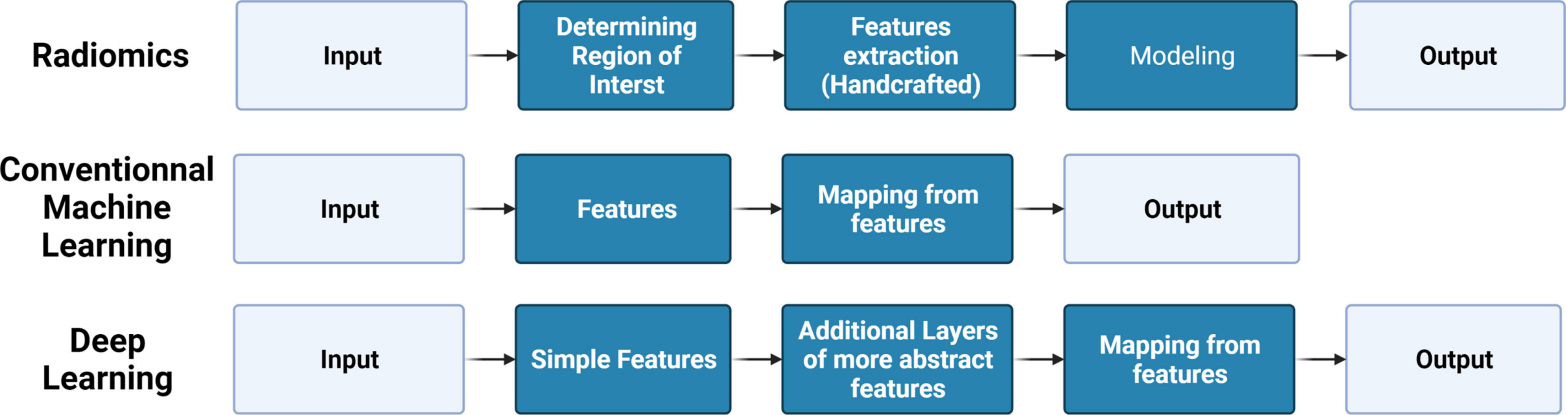
Brief comparison of the pipeline of radiomics, conventional machine learning, and deep learning.

In 1980, Fukushima (63) developed the neocognitron architecture to automatically identify the best features of an image, which is thought to be the prototype of a convolutional neural network (CNN). Convolution filters contained in CNNs can automatically extract features, while models with simple convolution layers can extract low-level visual information such as color, texture, and shape, and deeper convolution layers can extract abstract semantic information. Until 2012, a deep convolution network called AlexNet, proposed by Krizhevsky, had excellent performance in the ImageNet competition, providing a powerful method far surpassing the traditional method of manually designing features (64). Since then, the deep learning method has gradually become a hot topic in AI research. In 2017, Mishra et al. first introduced deep learning models (AlexNet, LeNet, and VGGNet) to improve the efficiency and accuracy of differentiating osteosarcoma tumor tissues and their counterparts (65). Since then, a substantial number of studies focused on deep learning in bone tumors have emerged in recent years. The relationships, development, and comparison of AI, ML, deep learning, radiomic, and deep learning in bone tumors are presented in detail in **Figures 1**, **2**.

## 3 Workflow for building a deep learning model

Deep learning technology enables the process of automatic feature extraction by learning deep features and relationship patterns of images directly from the architecture of multiple network layers on the original data (or preprocessed slightly) instead of manual extraction in the conventional ML method. In this way, the efficiency of image analysis is greatly improved without time-consuming manual feature extraction, and the system based on deep learning technology provides assistance for those without professional experience.

**Figure 3:**
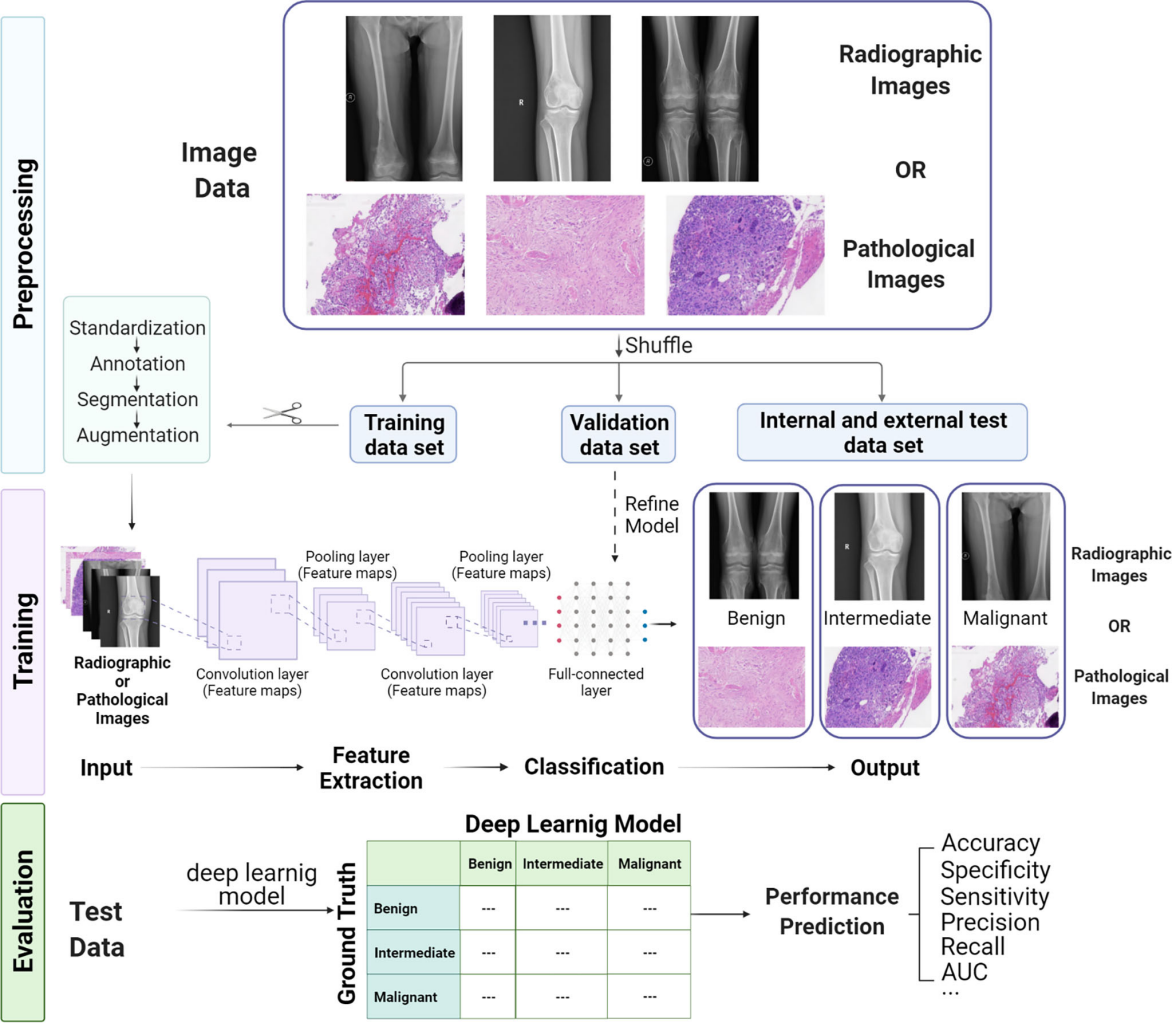
Workflow of building a deep learning model for bone tumor classification based on radiological and pathological images, including preprocessing, training, and evaluation.

Building a typical deep learning model for medical image analysis consists of three sections (**Figure 3**): (1) preprocessing images for sufficient quantity and suitable quality annotated data and splitting the dataset with an appropriate proportion (often 70%:20%:10% for training, validation and test dataset); (2) training the deep learning model on the training dataset and refining parameters; and (3) evaluating the performance of the model under the test dataset.

### 3.1 Image Preprocessing

Image preprocessing is a significant process to obtain a training dataset for building a deep learning model with high-quality and uniform format images. Since images may are collected from different instruments and through distinct acquisition settings, standardization of images can maintain data consistency and comparability for the same format as input. The detection network is utilized to show the area of interest on the image with a bounding box for segmentation, facilitating image mining and analysis. If there are not enough training images, image augmentation methods like digitally reconstructed radiographs (66–68) and generative adversarial networks (GANs) (69, 70) can be used to enlarge the image dataset. Remarkably, image labeling is the most time-consuming work because it requires researchers to manually annotate input–output pairs based on their clinical experience, but it is also the most critical step since these annotations are considered as “Gold Standard” for training and evaluation. The images obtained by the abovementioned methods are more suitable for model training than the original images and can significantly improve the performance of the model.

### 3.2 Model Training

CNNs are the most prevalently used multilayer neural network structures in image-based deep learning, and various CNN architectures like VGG, ResNet, and DenseNet have been used in medical image-based models. A typical CNN model consists of convolution layers, pooling layers, activation functions, and full-connected layers. In addition to batch standardization, dropout is also applied to optimize the performance of the model. Typically, in deep learning models based on CNN, there are three steps in data processing by the convolution layer. First, the convolution layer obtains a set of linear transformation outputs by multiple convolution calculations, and then each linear output is processed by a nonlinear activation function as a probe, and finally, the pooling function is used to further adjust the output of the convolution layer. It is important to note that the CNN model needs to be trained on a large amount of dataset for refining parameters. If the number of data is not enough, the model will overfit on the trained dataset and cannot be generalized in real data and will have poor performance.

### 3.3 Model Performance Evaluation

It is important to prepare a test dataset that has the same distribution as the training dataset. Running the trained model on the test dataset can provide an objective evaluation of model performance. There are many common evaluation metrics, including accuracy, precision, recall, sensitivity, specificity, the area under the curve (AUC) of the receiver operating characteristic (ROC) curve, and F1-score. Sometimes, we use single-number evaluation metrics like accuracy to simply compare different models, but most of the time, we utilize various metrics to evaluate models for a wide variety of uses. If we have to take both accuracy and running time into account, we can first define an “acceptable” running time, typically less than 100ms, and then maximize the classifier’s accuracy as much as possible within a limited running time frame. At this point, the running time represents the “satisfaction indicator,” and accuracy means the “optimization indicator.”

## 4 Deep learning applications in medical images for bone tumors

**Figure 4:**
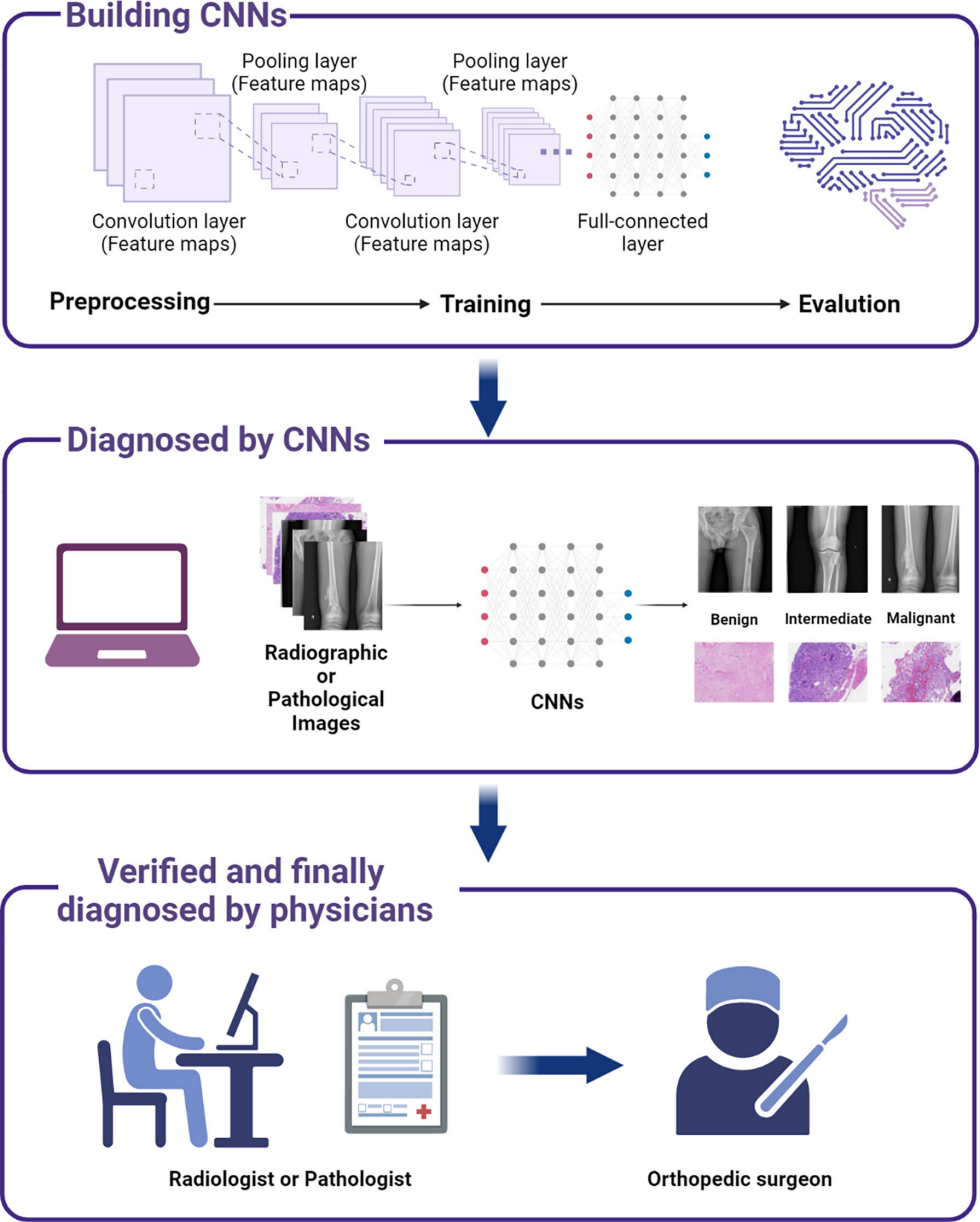
A scheme showing the process of incorporating deep learning models to assist bone tumor diagnosis and thereby facilitate decision making in clinical practice.

In the published studies of deep learning, multiple models were developed for analyzing radiological and pathological images, showing excellent performances that can be comparable to those of experienced physicians (including orthopedic surgeons, radiologists, and pathologists). Among them, the most analyzed radiological images for deep learning are generated from X-ray, CT, and MRI (71–80). These radiological images are used in deep learning for tumor detection and classification; differentiation of benign, intermediate, and malignant tumors; segmentation of the region of tumors; and tumor grading prediction. In addition, bone scintigraphy, PET, and spectral CT are also good tools to detect bone metastasis (81–93), as evidenced by their ability to identify the primary lesion of the tumor and calculate the volume of the metastatic sites, and applying deep learning to these radiological images can improve our diagnosis of bone metastasis. With regard to the pathological images, it is demonstrated that these features from resected tissues can reveal tumor histopathology after hematoxylin and eosin (H&E) staining and can also be used for tumor classification, prognosis prediction, and treatment guidance. Various models have been proposed to help clinicians diagnose and identify tumor regions on digital H&E-stained tissue images (65, 94–97). The details of the application of deep learning in primary and metastatic bone tumors are depicted in **Figure 4** and presented as follows.

### 4.1 Deep Learning in Primary Bone Tumors Based on Radiological Images

#### 4.1.1 Lesion Detection and Classification

X-ray, CT, and MR images are mainly used in deep learning for lesion detection and classification. Generated by X-ray, plain radiographs contain image parameters of tumor location, tumor size, and the margin of tumor for describing tumor characteristics. In 2020, He et al. (73) first developed a deep learning model based on CNN that could automatically classify primary bone tumors based on 2,899 plain radiographs from 1,356 patients using a multi-institutional dataset. The model had a high performance with an AUC reaching up to 0.877 and 0.916 for classifying benign and malignant, respectively, and with an accuracy of 72.1% for three-way classification (benign vs. intermediate vs. malignant), which was closed to 2 subspecialists, and outperformed junior radiologists. Similarly, combining global context and local patch for analysis, Do et al. (76) built a Multi-Level Seg-Unet model for the detection and classification of knee bone tumors on plain radiographs using 1,576 radiographs consisting of 1195 tumor images and 381 normal images with a superb accuracy of 99.05%.

Compared with plain radiographs, CT and MRI can provide further radiological information and improve lesion detection. Multiple deep learning methods have also been published for detecting and classifying bone tumors on CT and MRI (75, 77). For instance, a deep learning-based radiomics model (75) was described for discriminating between benign and malignant sacral tumors using 3D CT and clinical characteristics based on 1,316 manual-cropped radiomics features from 459 patients and achieved a high AUC of 0.83 in identifying benign and malignant sacral tumors. MRI is highly sensitive for the detection of bone abnormalities due to its ability to evaluate bone marrow involvement, soft tissue invasion, and fluid content of lesions (98, 99). A deep learning model (77) based on routine MRI and patient demographics using the EfficientNet-B0 architecture and a logistic regression model has also been described for identifying benign and malignant bone lesions, with an expert-level performance of accuracy, sensitivity, specificity, and AUC of 0.76, 0.79, 0.75, and 0.82, respectively.

#### 4.1.2 Segmentation and Volume Calculation

Another potential application of deep learning and radiological image analysis is the automatic segmentation and volume calculation of tumors (71, 72). Due to the large spatial and structural variabilities of tumors, osteosarcoma tumor segmentation on CT has been a challenging difficulty in AI. In 2017, Huang et al. (71) developed a multiple supervised fully convolutional networks (MSFCN) method to segment the region of tumors automatically. The deep end-to-end model used multiple feature channels to capture more context information and achieved an average DSC of 87.80%, an average sensitivity of 86.88%, an average HM of 19.81%, and an F1-measure of 0.908. A similar multiple supervised residual network (MSRN) model (72) based on ResNet cooperated with FCN and also has a high performance for osteosarcoma segmentation trained and tested on 1,900 CT images from 15 osteosarcoma patients. Furthermore, using a multiview fusion network to extract pseudo-3D information, a deep learning model (80) had the ability to perform the segmentation and volume calculation of pelvic bone tumors in MRI and reduce the average segmentation time by 100 times compared to other methods, which has a significant impact on the clinical practice of musculoskeletal oncology.

#### 4.1.3 Tumor Grading

Tumor grading is crucial for treatment plan making and prognosis prediction (100). A deep learning-based tumor grading model on T1-weighted or T2-weighted MRI sequences was proposed by Navarro et al. (79) in soft-tissue sarcoma patients. Grading the sample into low grade (G1) and high grade (G2/G3), the deep learning method achieved an F1-score of 0.90 and an AUC of 0.76. It provides insight for surgeons to make treatment plans while reducing invasive biopsies. Although there is currently no similar research in primary bone tumors, it may be a potential research direction in the near future.

#### 4.1.4 Tumor Necrosis Rate Assessment

Evaluation of tumor necrosis rate after neoadjuvant chemotherapy in patients with malignant bone tumors is critical since it can show the treatment response of patients and thus offer guidance for the subsequent chemotherapy after surgery (101). Currently, this process is assessed by multiple pathological slices, and it is so complex and time consuming that it limits its clinical practicality. Thus, it is necessary to convey other convenient and noninvasive methods to identify the tumor necrosis caused by neoadjuvant chemotherapy. To a great extent, it may facilitate our clinical evaluation of tumor chemotherapy sensitivity and help us classify patients into responders (≥90% tumor) and nonresponders (<90% necrosis) (102).

In 2020, a preliminary study conducted by Huang et al. applied ML to predict tumor necrosis rate on multiparametric MRI before and after chemotherapy in patients with osteosarcoma (103). This study is of great significance since it first explored the potential correlation between contrast-enhanced MRI and postoperative pathological features. More recently, other research performed by Kim et al. (104) first demonstrated that texture features of ML of positron emission tomography/computed tomography (PET/CT) images could reflect the fluorine-18fluorodeoxyglucose (18F-FDG) uptake heterogeneity features in osteosarcoma and therefore predict the treatment response to neoadjuvant chemotherapy. Although these studies used a supervised ML method instead of deep learning due to the small sample size, it is plausible and meaningful to expand the sample size and apply deep learning to predict histologic response by adopting other radiological imaging modalities (MRI, PET/CT), which may help clinicians to decide whether to continue the prior chemotherapy regimen to treat patients after surgery (104).

#### 4.1.5 Prognosis Prediction

The recurrent risk of bone tumors after surgery is a matter of great concern to orthopedic surgeons (105). Treatment for a bone tumor like curettage may be less invasive than en bloc wide resection. Meanwhile, the rates of recurrence and metastases are increased (2, 106). Thus, it is crucial to predict postsurgery regional recurrence of tumors based on presurgery medical images, which is not easy for physicians. Based on 56 patients with confirmed giant cell bone tumors (GCTB) in histopathology after curettage, a CNN model was reported by He et al. (107) to predict the local recurrence of GCTB on presurgery MRI. Without integrating any clinical characteristics, the pure image-based CNN model achieved an accuracy of 75.5% and a sensitivity of 85.7%. Using logistic regression, the fusion model was built by integrating tumor location, patient age, and CNN prediction, and the accuracy and sensitivity for prediction were improved to 78.6% and 87.5%, respectively, much higher than that of the radiologists (64.3% and 58.3%) (107). It is obvious that the regression deep learning model combined with clinical characteristics has a great potential to predict tumor recurrence and thereby guide clinical decision making.

### 4.2 Deep Learning in Bone Metastasis Based on Radiological Images

In recent years, various technologies have been used in the diagnosis of bone metastasis from different primary tumors, including bone scintigraphy, CT, MRI, and PET/CT (108, 109). The deep learning method is utilized in these modalities to detect the presence of bone metastasis, segment and calculate the volume of the metastatic lesions, differentiate the source of the primary tumors, and denoise scintigraphy to improve the quality of images.

Bone scintigraphy with 99mTc-MDP is widely applied in the detection and localization of bone metastasis in cancer patients since it has the merit of whole-body detection and high sensitivity for the diagnosis of bone metastasis (110–112). Several studies (86–90, 92, 93, 113–115) have explored applications of deep learning models in interpreting images of bone scintigraphy, and these models aid in diagnosing lesions and reducing the workload for clinical physicians. Papandrianos et al., (88, 89) proposed two deep learning models based on RGB-CNN architecture that can be trained efficiently on a small dataset and consume less running time to identify the bone metastasis and differentiate between a bone metastatic and a degenerative lesion in prostate cancer patients. Despite the models using small datasets (778 patients for model 1 and 507 patients for model 2) without any clinical information as input, the classification accuracy for two classes (bone metastasis or healthy) and three classes (normal, malignant, and degenerative) reached up to 97.38% and 91.42%, respectively, outperforming than other well-known CNN approaches. Cheng et al., (93) Cheng et al., (113) proposed CNN models to detect bone metastasis in the pelvis and identify metastasis spots on the ribs or spine in the chest from prostate cancer patients’ bone scintigraphy, with a sensitivity of 0.87 by using a hard positive mining (HPM) approach as an effective augmentation method and faster R-CNN and YOLO v3 to identify hotspots. Compared with analyzing only one image at a time, Pi et al. (90) developed a novel CNN approach to analyze both the anterior and posterior views of WBS examination for the presence of bone metastasis in various cancer patients and use a spatial attention feature aggregation operator for better spatial location information. Trained on 15,474 examinations from 13,811 patients, the model had excellent performance with an F1, accuracy, sensitivity, and specificity of 0.933, 95.00%, 93.17%, and 96.60%, respectively. Similarly, a multi-input CNN model (92) was designed for identifying bone metastasis in breast, prostate, lung, and other cancer patients based on a large number of bone scans (12,222 cases of bone scintigraphy). Using multiple images as input, the machine performed well, with the AUC of 0.98, 0.955, 0.957, and 0.971 for breast cancer, prostate cancer, lung cancer, and other cancers, respectively. It is noted that integrating local and global information would improve the performance of the model, Han et al. (114) proposed a 2D whole body-based and “global-local unified emphasis” (GLUE) CNN model for detecting bone metastasis on bone scans with a high accuracy of 0.900 and 0.889. In addition, some studies (86) utilized deep learning to denoise scintillation camera images, significantly promoting the ability of the model to detect bone metastasis in whole body bone scans by a Monte Carlo simulation approach.

Moreover, multiple deep learning methods have been used to identify bone metastasis on CT and MRI as well. In 2018, Chmelik et al. (82) reported a CNN-based method to classify and segment spine metastasis of lytic and sclerotic lesions in whole-spine CT scans with 1,046 lytic lesions and 1,135 sclerotic lesions from 31 cases. Trained on voxel-wise labeled images by two independent radiologists, the machine had 92% sensitivity in classifying and localizing small metastatic lesions greater than 1.4 mm^3^ under object-wise evaluation. Similarly, a deep learning-based DC-U-Net model (116) was also built to identify and segment spinal metastasis in lung cancer on spectral CT (dual-energy) images and have expert-level performance. Fan et al. (117) proposed a deep approach that employed the AdaBoost algorithm to classify images and the Chan-Vese (CV) algorithm to segment the lesion for the diagnosis of spinal bone metastasis in lung cancer patients on MRI images, achieving a high classification accuracy of 96.55%. Also, a deep learning model (115) using 2,880 annotated CT scans of 114 patients with prostate cancer was able to detect and classify bone lesions as benign and malignant, with a high accuracy of 92.2%. Volume calculation of metastatic lesions is important for clinical decision making. Lindgren Belal et al. (84) trained a CNN approach on 100 CT scans that was capable of segmenting and calculating the volume of the metastatic lesions authomatically with performance comparable to that of an experienced radiologist.

In addition to detecting bone metastasis, a deep learning model can also be applied to identify the origin of metastatic lesions. A DCE-MRI analysis method (83) based on deep learning was developed by Lang et al. (83) to detect bone metastasis in the spine and distinguish the tumor originating from primary lung cancer or other cancers. Compared with the methods based on hotspots or radiomics, the deep learning model had better performance for differentiating the origin of the metastatic lesions with an accuracy of 0.81, which would assist in predicting the primary cancer source when expansive PET/CT is not available.

### 4.3 Deep Learning in Bone Tumors Based on Pathological Images

Pathological images based on formalin-fixed paraffin-embedded (FFPE) tissues are often used as the gold standard for routine diagnosis of bone tumors. Tumor histopathology revealed by HE-stained tissue images and immunohistochemistry (IHC) for specific biomarkers can provide crucial information for the prediction of their clinical prognosis and sensitivity to therapy. In recent years, deep learning methods have been used to diagnose and identify tumor regions on digital H&E-stained tissue images as the reference standard.

To classify the region of viable and necrotic tumor in whole slide images (WSIs) of osteosarcoma, trained on 536 nontumor tiles, 263 necrotic tumor tiles, and 345 viable tumor tiles annotated by two pathologists, a CNN model was proposed as a classifier for differentiating osteosarcoma WSIs into a viable tumor, necrotic tumor, and nontumor in H&E-stained images with an accuracy of 91.2% (94). Tumor-prediction maps were utilized to visualize the tumor region and type classified by the CNN model, while they can also be used for calculating the percentage of the necrotic region and showing the area of the tumor over the WSIs. A similar CNN model was demonstrated by Mishra et al. with an accuracy of 92.4%, a precision of 97%, and an F1-score of 95%. By using the Siamese network and a new residual block called DRB, a DS-Net classifier composed of ASN and CN can utilize paired data as the input and refine the architecture through input pairs labeled by experts (95), and the model reached up to an average accuracy of 95.1% for the classification of viable and necrotic tumor regions in osteosarcoma. Moreover, a CNN classifier utilized by Foersch et al. (97) can also distinguish subtypes of soft tissue sarcoma from histopathological slides as well, and the accuracy of the pathologists was markedly improved from 46.3% to 87.1%.

In addition to tumor diagnosis assistance, the deep learning model cooperated with the application of a statistical approach for survival analysis and can also provide prognosis prediction of sarcoma (97). In leiomyosarcoma, CNN classifiers with the use of H&E-stained tissue images were shown to be useful for survival analysis with a mean AUC of 0.91 and an accuracy of 88.9%. In addition, class activation maps were used to visualize image regions and features that the model detected, showing that the prediction regions of “dead” were associated with fewer lymphoid infiltrates, more prominent intercellular matrix, presence of intratumoral hemorrhage, and more tumor-associated vessels, which are known as valuable prognostic factors for tumors (97).

## 5 Remaining limitations and future perspectives

Despite the application of various successful deep learning algorithms (details in **Table 1**), several limitations and potential perspectives need to be addressed as well.

**Table 1:** Summary of applications of deep learning-based artificial intelligence in bone tumors.

Authors (year)	Input feature	Applications	Deep learning methods	Size of dataset				Performance
				Dataset	Training	Validation	Testing	
He et al. (118)	X-ray	Classify primary bone tumors	CNN	2,899 images/1,356 patients	70%	10%	20%	Accuracy: 0.734 AUC: 0.877 (benign); 0.916 (malignant)
Do et al. (76)	X-ray	Detect, classify, and segment knee bone tumors	Multilevel Seg-Unet model	1,576 images	80%	20%	NA	Accuracy: 0.99 Average mean IoU: 0.848
Liu et al. (78)	X-ray; clinical characteristics	Classify benign, intermediate, and malignant tumors	CNN (Inception_v3)	982 images	784 images	97 images	101 images	AUC: 0.898 (benign); 0.894 (malignant); 0.865 (intermediate) Macroaverage AUC: 0.872
Huang et al. (71)	CT	Segment osteosarcoma	CNN (VGG-16); multiple supervised side output layers (MFSCN)	2,305 images/23 patients	1,900 images	NA	405 images	Sensitivity: 86.88% F1: 0.908 DSC: 87.80% Average HM: 19.81%
Yin et al. (75)	CT; clinical characteristics	Classify benign or malignant sacral tumors	DNN	1,316 images/459 patients	321 patients	138 patients	NA	Accuracy: 0.81 AUC: 0.84
Eweje et al. (77)	MRI; clinical characteristics	Classify benign and malignant bone lesions	CNN (EfficientNet)	1,060 images	70%	20%	10%	Accuracy: 0.76 Sensitivity: 0.79 Specificity: 0.75 AUC: 0.82
Papandrianos et al. (89)	Bone scintigraphy images	Identify the presence of bone metastasis in prostate cancer	CNN	778 patients	505 patients	156 patients	117 patients	Accuracy: 0.9142
Zhao et al. (92)	Bone scintigraphy images	Identify bone metastasis	CNN	12,222 patients	9,776 patients	1,223 patients	1,223 patients	AUC: 0.988 (breast cancer);.955 (prostate cancer);.957 (lung cancer);.971 (other cancers)
Papandrianos et al. (88)	Bone scintigraphy imaging	Classify malignant (bone metastasis) or healthy in prostate cancer	CNN	586 images	68%	17%	15%	Accuracy: 0.9738 Sensitivity: 0.965 Specificity: 0.968 Precision: 0.969 Recall: 0.974 F1: 0.97
Cheng et al. (93, 113)	Bone scintigraphy images	Identify bone metastases in the pelvis, ribs, or spinal cord	R-CNN; CNN (YOLO v3)	576 WBBS images: 205 prostate cancer/371 breast cancer	NA	NA	NA	Sensitivity: 0.82 for chest; 0.87 for pelvis Specificity: 0.81 for the pelvis Precision: 0.7 for the chest
Han et al. (114)	Bone scintigraphy images	Detect bone metastasis in prostate cancer	CNN	9,133 bone scans/5,342 patients	Abundant: 72% Limited: 10%	Abundant: 8% Limited: 40%	Abundant: 20% Limited: 50%	Accuracy: GLUE: 0.900; WB: 0.889 AUC: GLUE vs. WB: 0.894–0.908 vs. 0.870–0.877
Pi et al. (90)	Bone scintigraphy images	Identify bone metastasis	CNN; SDNN	15,474 images/13,811 patients	12,274 images	1,600 images	1,600 images	Accuracy: 0.95 Sensitivity: 0.9317 Specificity: 0.961 MSE: 0.933
Lang et al. (83)	DCE-MRI	Differentiate spinal metastases originating from lung and other cancers	Convolutional long short-term memory (CLSTM) network	61 patients	NA	NA	NA	Accuracy: 0.810
Masoudi et al. (115)	CT	Classify benign or malignant bone lesions in prostate cancer	CNN (2D ResNet-50; 3D ResNet-18)	2,880 CT scans/114 patients	75%	12%	13%	Accuracy: 92.2% F1: 92.3%
Fu et al. (95)	H&E slides	Classify viable and necrotic tumor regions in osteosarcoma	CNN (DS-Net)	1,144 images	60% (654)	20% (218)	20% (219)	Accuracy: 0.951 Sensitivity: 0.920 Specificity: 0.961 Precision: 0.929 F1: 0.922
Mishra et al. (65)	H&E slides	Classify tumor (viable tumor, necrosis) and nontumor region in osteosarcoma	CNN	82 WSIs/1,000 images	60%	20%	20%	Accuracy: 0.924 Precision: 0.970 Recall: 0.940 F1: 0.950
Arunachalam et al. (94)	H&E slides	Classify viable and necrotic tumor regions in osteosarcoma	CNN	40 WSIs/1,144 tiles	NA	NA	NA	Accuracy: 0.912

AI, artificial intelligence; AUC, area under the curve; CNN, Convolutional Neural Networks; CT, computed tomography; H&E, hematoxylin and eosin; MRI, magnetic resonance imaging; NA, not available; SDNN, Standard Deep Neural Network; WBBS, whole-body bone scan; WSI, whole slide images.

Firstly, training a deep learning model needs substantial labeled data. While image collection and annotation are the most time-consuming tasks, they need an experienced physician for image annotation since this labeling would be the gold standard for later training and testing. The quality of training datasets determines the model’s performance. However, it is pretty difficult to achieve in a bone tumor, which may be attributed to the following reasons: (1) the incidence and prevalence of bone tumors, such as osteosarcoma, are pretty low, thus the number of cases is comparatively small, and most of the data are concentrated in large teaching hospitals; (2) it is expensive and sometimes unrealistic to require experienced physicians to spend much time in labeling images with high quality; (3) medical images involve patient privacy, and in the ethically demanding medical field, it is difficult to obtain the permission to use of large-scale data.

Secondly, there has been significant progress in data collection and sharing in recent years. A number of public datasets like TCGA (119), CCC-EMN, and Cancer Genome Atlas Research Network (120) for cancers have been published, and several methods using public datasets achieved good performances. Unfortunately, data concerning bone tumors are absent in most available public databases, creating obstacles to conducting deep learning in this field. However, transfer learning refers to training models on other abundant medical images and transferring them to target images, which may be a good tool since the amount of data and required time will be greatly minimized when learning new tasks (121, 122).

Thirdly, most published models were designed to deal with a single task. However, in clinical practice, physicians do not rely on a single medical file for a final diagnosis. For instance, physicians need to incorporate the clinical parameters and radiological and pathological images to achieve the diagnosis of osteosarcoma. Furthermore, in pathological diagnosis, the pathologists also need to take into account the results of other IHC biomarkers, such as Ki-67, SATB2, and MDM2, or even next-generation sequencing (NGS), to reach a confirmed diagnosis in addition to H&E staining (123–126). If the models are built based on a single parameter, their clinical value may be greatly jeopardized. Therefore, more comprehensive models combining various characteristics should be designed and emphasized in the future.

Fourthly, primary bone tumors and tumor bone metastases were mentioned above, while studies regarding lung metastasis of malignant bone tumors, the main cause of death in sarcoma patients, have not been explored yet. Early identification of lung metastasis is critical for alleviating prognosis and treatment of patients (127). Recently, deep learning approaches have been applied to detect and classify lung metastasis and build deep learning-based image reconstruction techniques (128, 129) that can significantly reduce the radiation dose of CT (103). Due to the crucial role of lung metastasis detection for prognosis in malignant bone tumors, more efficient models are expected to be developed.

Fifthly, the genetic information of tumors can be used to predict tumor subtypes and the prognosis of patients, but the related profiling remains costly. In recent years, gene expression prediction based on deep learning models has become a research hotspot with the development of high-throughput sequencing as well as the application of deep learning in genomics and transcriptomics. Several models based on deep learning in gene expression prediction have shown good performance in several tumors (125), such as lung cancer (130–132) and breast cancer (73, 133). However, the relevant studies have not been performed on a bone tumor, and it has gained a promising expectation to apply deep learning for the prediction of gene expression in the bone tumor.

Lastly, spectroscopy, such as Raman spectroscopy, can provide quantifiable and label-free information for molecular patterns of diseases and can be combined with deep learning to identify and diagnose tumors, which is a novel and promising field. Currently, there are several models using spectroscopy to predict several types of tumors, such as pancreatic cancer (134), nasopharyngeal cancer (135), breast cancer (118, 136), lung cancer (137), and tongue cancer (138). However, the combination of spectroscopy and deep learning is scarce. Only one CNN model (91) based on 1,281 serum Raman spectra from 427 patients was reported to identify prostate cancer bone metastases with high performance, and there is no related study in primary bone tumors. Therefore, it is promising to develop deep learning approaches based on spectroscopy with or without radiological/pathological images to pave a new avenue for the identification of bone tumors.

## 6 Conclusion

In conclusion, there have been various studies using deep learning methods in bone tumor diagnosis on radiological and pathological images. Many of them have shown excellent performances in detection, classification, segmentation, and volume calculation of the primary tumor and bone metastasis. In addition, some models taking other clinical characteristics into consideration were proposed to predict the prognosis of cancers with even higher accuracy, and the prediction of the deep learning model was demonstrated as an independent prognosis factor. However, there are few models that have been really applied in clinical workflow, which may be attributed to their poor generalization capability to clinical practice. Currently, the suitable training data are insufficient and most models can only process a single data, opposing barriers to its implementation. Future research may deal with these limitations and focus on a more diverse way to implement deep learning models into clinical practice. Generally, deep learning methods could provide powerful assistance for clinicians and reduce time consumption and economic burden while optimizing clinical treatment strategies in bone tumors.

## Author Contributions

XZ: investigation, data analysis, and visualization, writing—original draft preparation. HW, CF, RX, YH, and LL: investigation and editing. CT: conceptualization, supervision, writing, and revision. All authors have read and agreed to the published version of the manuscript.

## Funding

This work was supported by the National Natural Foundation of China (81902745), Hunan Provincial Natural Science Foundation of China (2022JJ30843), the Science and Technology Development Fund Guided by Central Government (2021Szvup169), Hunan Provincial Administration of Traditional Chinese Medicine Project (No. D2022117), and the Clinical Research Center for Medical Imaging in Hunan Province (2020SK4001).

## Conflict of Interest

The authors declare that the research was conducted in the absence of any commercial or financial relationships that could be construed as a potential conflict of interest.

## Publisher’s Note

All claims expressed in this article are solely those of the authors and do not necessarily represent those of their affiliated organizations, or those of the publisher, the editors and the reviewers. Any product that may be evaluated in this article, or claim that may be made by its manufacturer, is not guaranteed or endorsed by the publisher.

## References

[1] Ottaviani G Jaffe N. The Epidemiology of Osteosarcoma. Cancer Treat Res (2009) 152:3–13. doi: 10.1007/978-1-4419-0284-9_1 20213383

[2] Ritter J Bielack SS. Osteosarcoma. Ann Oncol (2010) 21 Suppl 7:vii320–325. doi: 10.1093/annonc/mdq276 20943636

[3] Tu C He J Chen R Li Z. The Emerging Role of Exosomal Non-Coding RNAs in Musculoskeletal Diseases. Curr Pharm Des (2019) 25(42):4523–35. doi: 10.2174/1381612825666191113104946 31724510

[4] Sheng G Gao Y Yang Y Wu H. Osteosarcoma and Metastasis. Front Oncol (2021) 11:780264. doi: 10.3389/fonc.2021.780264 34956899 PMC8702962

[5] Tu C He J Qi L Ren X Zhang C Duan Z. Emerging Landscape of Circular RNAs as Biomarkers and Pivotal Regulators in Osteosarcoma. J Cell Physiol (2020) 235(12):9037–58. doi: 10.1002/jcp.29754 32452026

[6] Tu C Yang K Wan L He J Qi L Wang W. The Crosstalk Between lncRNAs and the Hippo Signalling Pathway in Cancer Progression. Cell Prolif. (2020) 53(9):e12887. doi: 10.1111/cpr.12887 32779318 PMC7507458

[7] He J Ling L Liu Z Ren X Wan L Tu C. Functional Interplay Between Long non-Coding RNAs and the Wnt Signaling Cascade in Osteosarcoma. Cancer Cell Int (2021) 21(1):313. doi: 10.1186/s12935-021-02013-8 34130697 PMC8207720

[8] Zhang C He J Qi L Wan L Wang W Tu C. Diagnostic and Prognostic Significance of Dysregulated Expression of Circular RNAs in Osteosarcoma. Expert Rev Mol Diagn (2021) 21(2):235–44. doi: 10.1080/14737159.2021.1874922 33428501

[9] Ferguson JL Turner SP. Bone Cancer: Diagnosis and Treatment Principles. Am Fam. Physician (2018) 98(4):205–13. 30215968

[10] Leite TC Watters RJ Weiss KR Intini G. Avenues of Research in Dietary Interventions to Target Tumor Metabolism in Osteosarcoma. J Transl Med (2021) 19(1):450. doi: 10.1186/s12967-021-03122-8 34715874 PMC8555297

[11] Li W Ding Z Wang D Li C Pan Y Zhao Y. Ten-Gene Signature Reveals the Significance of Clinical Prognosis and Immuno-Correlation of Osteosarcoma and Study on Novel Skeleton Inhibitors Regarding MMP9. Cancer Cell Int (2021) 21(1):377. doi: 10.1186/s12935-021-02041-4 34261456 PMC8281696

[12] Ogrinc N Caux PD Robin YM Bouchaert E Fatou B Ziskind M. Direct Water-Assisted Laser Desorption/Ionization Mass Spectrometry Lipidomic Analysis and Classification of Formalin-Fixed Paraffin-Embedded Sarcoma Tissues Without Dewaxing. Clin Chem (2021) 67(11):1513–23. doi: 10.1093/clinchem/hvab160 34586394

[13] Hong L Cheng X Zheng D. Application of Artificial Intelligence in Emergency Nursing of Patients With Chronic Obstructive Pulmonary Disease. Contrast Media Mol Imaging (2021) 2021:6423398. doi: 10.1155/2021/6423398 34908913 PMC8635944

[14] Wu TT Zheng RF Lin ZZ Gong HR Li H. A Machine Learning Model to Predict Critical Care Outcomes in Patient With Chest Pain Visiting the Emergency Department. BMC Emerg Med (2021) 21(1):112. doi: 10.1186/s12873-021-00501-8 34620086 PMC8496015

[15] Kermany DS Goldbaum M Cai W Valentim CCS Liang H Baxter SL. Identifying Medical Diagnoses and Treatable Diseases by Image-Based Deep Learning. Cell (2018) 172(5):1122–1131.e1129. doi: 10.1016/j.cell.2018.02.010 29474911

[16] Gulshan V Peng L Coram M Stumpe MC Wu D Narayanaswamy A. Development and Validation of a Deep Learning Algorithm for Detection of Diabetic Retinopathy in Retinal Fundus Photographs. Jama (2016) 316(22):2402–10. doi: 10.1001/jama.2016.17216 27898976

[17] Ardila D Kiraly AP Bharadwaj S Choi B Reicher JJ Peng L. End-To-End Lung Cancer Screening With Three-Dimensional Deep Learning on Low-Dose Chest Computed Tomography. Nat Med (2019) 25(6):954–61. doi: 10.1038/s41591-019-0447-x 31110349

[18] Munir K Elahi H Ayub A Frezza F Rizzi A. Cancer Diagnosis Using Deep Learning: A Bibliographic Review. Cancers (Basel) (2019) 11(9):908873. doi: 10.3390/cancers11091235 PMC6770116 31450799

[19] Raman R Srinivasan S Virmani S Sivaprasad S Rao C Rajalakshmi R. Fundus Photograph-Based Deep Learning Algorithms in Detecting Diabetic Retinopathy. Eye (Lond) (2019) 33(1):97–109. doi: 10.1038/s41433-018-0269-y 30401899 PMC6328553

[20] Schelb P Kohl S Radtke JP Wiesenfarth M Kickingereder P Bickelhaupt S. Classification of Cancer at Prostate MRI: Deep Learning Versus Clinical PI-RADS Assessment. Radiology (2019) 293(3):607–17. doi: 10.1148/radiol.2019190938 31592731

[21] Wang S Shi J Ye Z Dong D Yu D Zhou M. Predicting EGFR Mutation Status in Lung Adenocarcinoma on Computed Tomography Image Using Deep Learning. Eur Respir J (2019) 53(3):1800986. doi: 10.1183/13993003.00986-2018 30635290 PMC6437603

[22] Xu Y Hosny A Zeleznik R Parmar C Coroller T Franco I. Deep Learning Predicts Lung Cancer Treatment Response From Serial Medical Imaging. Clin Cancer Res (2019) 25(11):3266–75. doi: 10.1158/1078-0432.Ccr-18-2495 PMC6548658 31010833

[23] AlDubayan SH Conway JR Camp SY Witkowski L Kofman E Reardon B. Detection of Pathogenic Variants With Germline Genetic Testing Using Deep Learning vs Standard Methods in Patients With Prostate Cancer and Melanoma. Jama (2020) 324(19):1957–69. doi: 10.1001/jama.2020.20457 PMC7672519 33201204

[24] Bulten W Pinckaers H van Boven H Vink R de Bel T van Ginneken B. Automated Deep-Learning System for Gleason Grading of Prostate Cancer Using Biopsies: A Diagnostic Study. Lancet Oncol (2020) 21(2):233–41. doi: 10.1016/s1470-2045(19)30739-9 31926805

[25] Kido S Hirano Y Mabu S. Deep Learning for Pulmonary Image Analysis: Classification, Detection, and Segmentation. Adv Exp Med Biol (2020) 1213:47–58. doi: 10.1007/978-3-030-33128-3_3 32030662

[26] Zhang M Young GS Chen H Li J Qin L McFaline-Figueroa JR. Deep-Learning Detection of Cancer Metastases to the Brain on MRI. J Magn Reson Imaging (2020) 52(4):1227–36. doi: 10.1002/jmri.27129 PMC7487020 32167652

[27] Chung SW Han SS Lee JW Oh KS Kim NR Yoon JP. Automated Detection and Classification of the Proximal Humerus Fracture by Using Deep Learning Algorithm. Acta Orthop. (2018) 89(4):468–73. doi: 10.1080/17453674.2018.1453714 PMC6066766 29577791

[28] Kim DH MacKinnon T. Artificial Intelligence in Fracture Detection: Transfer Learning From Deep Convolutional Neural Networks. Clin Radiol (2018) 73(5):439–45. doi: 10.1016/j.crad.2017.11.015 29269036

[29] Tomita N Cheung YY Hassanpour S. Deep Neural Networks for Automatic Detection of Osteoporotic Vertebral Fractures on CT Scans. Comput Biol Med (2018) 98:8–15. doi: 10.1016/j.compbiomed.2018.05.011 29758455

[30] Cheng CT Ho TY Lee TY Chang CC Chou CC Chen CC. Application of a Deep Learning Algorithm for Detection and Visualization of Hip Fractures on Plain Pelvic Radiographs. Eur Radiol (2019) 29(10):5469–77. doi: 10.1007/s00330-019-06167-y PMC6717182 30937588

[31] Derkatch S Kirby C Kimelman D Jozani MJ Davidson JM Leslie WD. Identification of Vertebral Fractures by Convolutional Neural Networks to Predict Nonvertebral and Hip Fractures: A Registry-Based Cohort Study of Dual X-Ray Absorptiometry. Radiology (2019) 293(2):405–11. doi: 10.1148/radiol.2019190201 31526255

[32] Gan K Xu D Lin Y Shen Y Zhang T Hu K. Artificial Intelligence Detection of Distal Radius Fractures: A Comparison Between the Convolutional Neural Network and Professional Assessments. Acta Orthop. (2019) 90(4):394–400. doi: 10.1080/17453674.2019.1600125 30942136 PMC6718190

[33] Kitamura G Chung CY Moore BE 2nd. Ankle Fracture Detection Utilizing a Convolutional Neural Network Ensemble Implemented With a Small Sample, *De Novo* Training, and Multiview Incorporation. J Digit Imaging (2019) 32(4):672–7. doi: 10.1007/s10278-018-0167-7 PMC6646476 31001713

[34] Pranata YD Wang KC Wang JC Idram I Lai JY Liu JW. Deep Learning and SURF for Automated Classification and Detection of Calcaneus Fractures in CT Images. Comput Methods Prog. BioMed (2019) 171:27–37. doi: 10.1016/j.cmpb.2019.02.006 30902248

[35] Blüthgen C Becker AS Vittoria de Martini I Meier A Martini K Frauenfelder T. Detection and Localization of Distal Radius Fractures: Deep Learning System Versus Radiologists. Eur J Radiol (2020) 126:108925. doi: 10.1016/j.ejrad.2020.108925 32193036

[36] Jones RM Sharma A Hotchkiss R Sperling JW Hamburger J Ledig C. Assessment of a Deep-Learning System for Fracture Detection in Musculoskeletal Radiographs. NPJ Digit Med (2020) 3:144. doi: 10.1038/s41746-020-00352-w 33145440 PMC7599208

[37] Štajduhar I Mamula M Miletić D Ünal G. Semi-Automated Detection of Anterior Cruciate Ligament Injury From MRI. Comput Methods Prog. BioMed (2017) 140:151–64. doi: 10.1016/j.cmpb.2016.12.006 28254071

[38] Bien N Rajpurkar P Ball RL Irvin J Park A Jones E. Deep-Learning-Assisted Diagnosis for Knee Magnetic Resonance Imaging: Development and Retrospective Validation of MRNet. PloS Med (2018) 15(11):e1002699. doi: 10.1371/journal.pmed.1002699 30481176 PMC6258509

[39] Liu F Zhou Z Samsonov A Blankenbaker D Larison W Kanarek A. Deep Learning Approach for Evaluating Knee MR Images: Achieving High Diagnostic Performance for Cartilage Lesion Detection. Radiology (2018) 289(1):160–9. doi: 10.1148/radiol.2018172986 PMC6166867 30063195

[40] Tiulpin A Thevenot J Rahtu E Lehenkari P Saarakkala S. Automatic Knee Osteoarthritis Diagnosis From Plain Radiographs: A Deep Learning-Based Approach. Sci Rep (2018) 8(1):1727. doi: 10.1038/s41598-018-20132-7 29379060 PMC5789045

[41] Chang PD Wong TT Rasiej MJ. Deep Learning for Detection of Complete Anterior Cruciate Ligament Tear. J Digit Imaging (2019) 32(6):980–6. doi: 10.1007/s10278-019-00193-4 PMC6841825 30859341

[42] Germann C Marbach G Civardi F Fucentese SF Fritz J Sutter R. Deep Convolutional Neural Network-Based Diagnosis of Anterior Cruciate Ligament Tears: Performance Comparison of Homogenous Versus Heterogeneous Knee MRI Cohorts With Different Pulse Sequence Protocols and 1.5-T and 3-T Magnetic Field Strengths. Invest Radiol (2020) 55(8):499–506. doi: 10.1097/rli.0000000000000664 32168039 PMC7343178

[43] Xue Y Zhang R Deng Y Chen K Jiang T. A Preliminary Examination of the Diagnostic Value of Deep Learning in Hip Osteoarthritis. PloS One (2017) 12(6):e0178992. doi: 10.1371/journal.pone.0178992 28575070 PMC5456368

[44] Norman B Pedoia V Noworolski A Link TM Majumdar S. Applying Densely Connected Convolutional Neural Networks for Staging Osteoarthritis Severity From Plain Radiographs. J Digit Imaging (2019) 32(3):471–7. doi: 10.1007/s10278-018-0098-3 PMC6499841 30306418

[45] Pedoia V Lee J Norman B Link TM Majumdar S. Diagnosing Osteoarthritis From T(2) Maps Using Deep Learning: An Analysis of the Entire Osteoarthritis Initiative Baseline Cohort. Osteoarthr. Cartilage (2019) 27(7):1002–10. doi: 10.1016/j.joca.2019.02.800 PMC6579664 30905742

[46] Guan B Liu F Mizaian AH Demehri S Samsonov A Guermazi A. Deep Learning Approach to Predict Pain Progression in Knee Osteoarthritis. Skeletal Radiol (2021) 51(2):363–73. doi: 10.1007/s00256-021-03773-0 PMC9232386 33835240

[47] Razmjoo A Caliva F Lee J Liu F Joseph GB Link TM. T(2) Analysis of the Entire Osteoarthritis Initiative Dataset. J Orthop. Res (2021) 39(1):74–85. doi: 10.1002/jor.24811 32691905

[48] Jamaludin A Lootus M Kadir T Zisserman A Urban J Battié MC. ISSLS PRIZE IN BIOENGINEERING SCIENCE 2017: Automation of Reading of Radiological Features From Magnetic Resonance Images (MRIs) of the Lumbar Spine Without Human Intervention is Comparable With an Expert Radiologist. Eur Spine J (2017) 26(5):1374–83. doi: 10.1007/s00586-017-4956-3 28168339

[49] Balsiger F Steindel C Arn M Wagner B Grunder L El-Koussy M. Segmentation of Peripheral Nerves From Magnetic Resonance Neurography: A Fully-Automatic, Deep Learning-Based Approach. Front Neurol (2018) 9:777. doi: 10.3389/fneur.2018.00777 30283397 PMC6156270

[50] Spampinato C Palazzo S Giordano D Aldinucci M Leonardi R. Deep Learning for Automated Skeletal Bone Age Assessment in X-Ray Images. Med Image Anal (2017) 36:41–51. doi: 10.1016/j.media.2016.10.010 27816861

[51] Koitka S Demircioglu A Kim MS Friedrich CM Nensa F. Ossification Area Localization in Pediatric Hand Radiographs Using Deep Neural Networks for Object Detection. PloS One (2018) 13(11):e0207496. doi: 10.1371/journal.pone.0207496 30444906 PMC6239319

[52] Larson DB Chen MC Lungren MP Halabi SS Stence NV Langlotz CP. Performance of a Deep-Learning Neural Network Model in Assessing Skeletal Maturity on Pediatric Hand Radiographs. Radiology (2018) 287(1):313–22. doi: 10.1148/radiol.2017170236 29095675

[53] Tong C Liang B Li J Zheng Z. A Deep Automated Skeletal Bone Age Assessment Model With Heterogeneous Features Learning. J Med Syst (2018) 42(12):249. doi: 10.1007/s10916-018-1091-6 30390162

[54] Ren X Li T Yang X Wang S Ahmad S Xiang L. Regression Convolutional Neural Network for Automated Pediatric Bone Age Assessment From Hand Radiograph. IEEE J BioMed Health Inform (2019) 23(5):2030–8. doi: 10.1109/jbhi.2018.2876916 30346295

[55] Gao Y Zhu T Xu X. Bone Age Assessment Based on Deep Convolution Neural Network Incorporated With Segmentation. Int J Comput Assist Radiol Surg (2020) 15(12):1951–62. doi: 10.1007/s11548-020-02266-0 32986142

[56] Yune S Lee H Kim M Tajmir SH Gee MS Do S. Beyond Human Perception: Sexual Dimorphism in Hand and Wrist Radiographs Is Discernible by a Deep Learning Model. J Digit Imaging (2019) 32(4):665–71. doi: 10.1007/s10278-018-0148-x PMC6646498 30478479

[57] Gillies RJ Kinahan PE Hricak H. Radiomics: Images Are More Than Pictures, They Are Data. Radiology (2016) 278(2):563–77. doi: 10.1148/radiol.2015151169 PMC4734157 26579733

[58] Abdel Razek AAK Alksas A Shehata M AbdelKhalek A Abdel Baky K El-Baz A. Clinical Applications of Artificial Intelligence and Radiomics in Neuro-Oncology Imaging. Insights Imaging (2021) 12(1):152. doi: 10.1186/s13244-021-01102-6 34676470 PMC8531173

[59] Kann BH Hosny A Aerts H. Artificial Intelligence for Clinical Oncology. Cancer Cell (2021) 39(7):916–27. doi: 10.1016/j.ccell.2021.04.002 PMC8282694 33930310

[60] Lalehzarian SP Gowd AK Liu JN. Machine Learning in Orthopaedic Surgery. World J Orthop. (2021) 12(9):685–99. doi: 10.5312/wjo.v12.i9.685 PMC8472446 34631452

[61] Turkbey B Haider MA. Deep Learning-Based Artificial Intelligence Applications in Prostate MRI: Brief Summary. Br J Radiol (2021) 95(1131):20210563. doi: 10.1259/bjr.20210563 34860562 PMC8978238

[62] Shin HC Roth HR Gao M Lu L Xu Z Nogues I. Deep Convolutional Neural Networks for Computer-Aided Detection: CNN Architectures, Dataset Characteristics and Transfer Learning. IEEE Trans Med Imaging (2016) 35(5):1285–98. doi: 10.1109/TMI.2016.2528162 PMC4890616 26886976

[63] Fukushima K. Neocognitron: A Self Organizing Neural Network Model for a Mechanism of Pattern Recognition Unaffected by Shift in Position. Biol Cybern (1980) 36(4):193–202. doi: 10.1007/bf00344251 7370364

[64] Schmidhuber J. Deep Learning in Neural Networks: An Overview. Neural Networks (2015) 61:85–117. doi: 10.1016/j.neunet.2014.09.003 25462637

[65] Mishra R Daescu O Leavey P Rakheja D Sengupta A. Convolutional Neural Network for Histopathological Analysis of Osteosarcoma. J Comput Biol (2018) 25(3):313–25. doi: 10.1089/cmb.2017.0153 29083930

[66] Mutasa S Varada S Goel A Wong TT Rasiej MJ. Advanced Deep Learning Techniques Applied to Automated Femoral Neck Fracture Detection and Classification. J Digit Imaging (2020) 33(5):1209–17. doi: 10.1007/s10278-020-00364-8 PMC7572965 32583277

[67] Almeida DF Astudillo P Vandermeulen D. Three-Dimensional Image Volumes From Two-Dimensional Digitally Reconstructed Radiographs: A Deep Learning Approach in Lower Limb CT Scans. Med Phys (2021) 48(5):2448–57. doi: 10.1002/mp.14835 33690903

[68] Mortani Barbosa EJ Jr. Gefter WB Ghesu FC Liu S Mailhe B Mansoor A. Automated Detection and Quantification of COVID-19 Airspace Disease on Chest Radiographs: A Novel Approach Achieving Expert Radiologist-Level Performance Using a Deep Convolutional Neural Network Trained on Digital Reconstructed Radiographs From Computed Tomography-Derived Ground Truth. Invest Radiol (2021) 56(8):471–9. doi: 10.1097/rli.0000000000000763 33481459

[69] Rubin M Stein O Turko NA Nygate Y Roitshtain D Karako L. TOP-GAN: Stain-Free Cancer Cell Classification Using Deep Learning With a Small Training Set. Med Image Anal (2019) 57:176–85. doi: 10.1016/j.media.2019.06.014 31325721

[70] Tembine H. Deep Learning Meets Game Theory: Bregman-Based Algorithms for Interactive Deep Generative Adversarial Networks. IEEE Trans Cybern (2020) 50(3):1132–45. doi: 10.1109/tcyb.2018.2886238 30605115

[71] Huang L Xia W Zhang B Qiu B Gao X. MSFCN-Multiple Supervised Fully Convolutional Networks for the Osteosarcoma Segmentation of CT Images. Comput Methods Prog. BioMed (2017) 143:67–74. doi: 10.1016/j.cmpb.2017.02.013 28391820

[72] Zhang R Huang L Xia W Zhang B Qiu B Gao X. Multiple Supervised Residual Network for Osteosarcoma Segmentation in CT Images. Comput Med Imaging Graph (2018) 63:1–8. doi: 10.1016/j.compmedimag.2018.01.006 29361340

[73] He B Bergenstrahle L Stenbeck L Abid A Andersson A Borg A. Integrating Spatial Gene Expression and Breast Tumour Morphology *via* Deep Learning. Nat BioMed Eng (2020) 4(8):827–34. doi: 10.1038/s41551-020-0578-x 32572199

[74] He Y Pan I Bao B Halsey K Chang M Liu H. Deep Learning-Based Classification of Primary Bone Tumors on Radiographs: A Preliminary Study. EBioMedicine (2020) 62:103121. doi: 10.1016/j.ebiom.2020.103121 33232868 PMC7689511

[75] Yin P Mao N Chen H Sun C Wang S Liu X. Machine and Deep Learning Based Radiomics Models for Preoperative Prediction of Benign and Malignant Sacral Tumors. Front Oncol (2020) 10:564725. doi: 10.3389/fonc.2020.564725 33178593 PMC7596901

[76] Do NT Jung ST Yang HJ Kim SH. Multi-Level Seg-Unet Model With Global and Patch-Based X-Ray Images for Knee Bone Tumor Detection. Diagn. (Basel) (2021) 11(4):691. doi: 10.3390/diagnostics11040691 PMC8070216 33924426

[77] Eweje FR Bao B Wu J Dalal D Liao WH He Y. Deep Learning for Classification of Bone Lesions on Routine MRI. EBioMedicine (2021) 68:103402. doi: 10.1016/j.ebiom.2021.103402 34098339 PMC8190437

[78] Liu R Pan D Xu Y Zeng H He Z Lin J. A Deep Learning-Machine Learning Fusion Approach for the Classification of Benign, Malignant, and Intermediate Bone Tumors. Eur Radiol (2021) 32(2):1371–83. doi: 10.1007/s00330-021-08195-z 34432121

[79] Navarro F Dapper H Asadpour R Knebel C Spraker MB Schwarze V. Development and External Validation of Deep-Learning-Based Tumor Grading Models in Soft-Tissue Sarcoma Patients Using MR Imaging. Cancers (Basel) (2021) 13(12):2866. doi: 10.3390/cancers13122866 34201251 PMC8227009

[80] Qu Y Li X Yan Z Zhao L Zhang L Liu C. Surgical Planning of Pelvic Tumor Using Multi-View CNN With Relation-Context Representation Learning. Med Image Anal (2021) 69:101954. doi: 10.1016/j.media.2020.101954 33550006

[81] Wang J Fang Z Lang N Yuan H Su MY Baldi P. A Multi-Resolution Approach for Spinal Metastasis Detection Using Deep Siamese Neural Networks. Comput Biol Med (2017) 84:137–46. doi: 10.1016/j.compbiomed.2017.03.024 PMC6042511 28364643

[82] Chmelik J Jakubicek R Walek P Jan J Ourednicek P Lambert L. Deep Convolutional Neural Network-Based Segmentation and Classification of Difficult to Define Metastatic Spinal Lesions in 3D CT Data. Med Image Anal (2018) 49:76–88. doi: 10.1016/j.media.2018.07.008 30114549

[83] Lang N Zhang Y Zhang E Zhang J Chow D Chang P. Differentiation of Spinal Metastases Originated From Lung and Other Cancers Using Radiomics and Deep Learning Based on DCE-MRI. Magn Reson Imaging (2019) 64:4–12. doi: 10.1016/j.mri.2019.02.013 30826448 PMC6713616

[84] Lindgren Belal S Sadik M Kaboteh R Enqvist O Ulén J Poulsen MH. Deep Learning for Segmentation of 49 Selected Bones in CT Scans: First Step in Automated PET/CT-Based 3D Quantification of Skeletal Metastases. Eur J Radiol (2019) 113:89–95. doi: 10.1016/j.ejrad.2019.01.028 30927965

[85] Lin Q Luo M Gao R Li T Man Z Cao Y. Deep Learning Based Automatic Segmentation of Metastasis Hotspots in Thorax Bone SPECT Images. PloS One (2020) 15(12):e0243253. doi: 10.1371/journal.pone.0243253 33270746 PMC7714246

[86] Minarik D Enqvist O Trägårdh E. Denoising of Scintillation Camera Images Using a Deep Convolutional Neural Network: A Monte Carlo Simulation Approach. J Nucl Med (2020) 61(2):298–303. doi: 10.2967/jnumed.119.226613 31324711 PMC8801959

[87] Ntakolia C Diamantis DE Papandrianos N Moustakidis S Papageorgiou EI. A Lightweight Convolutional Neural Network Architecture Applied for Bone Metastasis Classification in Nuclear Medicine: A Case Study on Prostate Cancer Patients. Healthc. (Basel) (2020) 8(4):493. doi: 10.3390/healthcare8040493 PMC7711827 33217973

[88] Papandrianos N Papageorgiou E Anagnostis A Papageorgiou K. Bone Metastasis Classification Using Whole Body Images From Prostate Cancer Patients Based on Convolutional Neural Networks Application. PloS One (2020) 15(8):e0237213. doi: 10.1371/journal.pone.0237213 32797099 PMC7428190

[89] Papandrianos N Papageorgiou EI Anagnostis A. Development of Convolutional Neural Networks to Identify Bone Metastasis for Prostate Cancer Patients in Bone Scintigraphy. Ann Nucl Med (2020) 34(11):824–32. doi: 10.1007/s12149-020-01510-6 32839920

[90] Pi Y Zhao Z Xiang Y Li Y Cai H Yi Z. Automated Diagnosis of Bone Metastasis Based on Multi-View Bone Scans Using Attention-Augmented Deep Neural Networks. Med Image Anal (2020) 65:101784. doi: 10.1016/j.media.2020.101784 32763793

[91] Shao X Zhang H Wang Y Qian H Zhu Y Dong B. Deep Convolutional Neural Networks Combine Raman Spectral Signature of Serum for Prostate Cancer Bone Metastases Screening. Nanomedicine (2020) 29:102245. doi: 10.1016/j.nano.2020.102245 32592757

[92] Zhao Z Pi Y Jiang L Xiang Y Wei J Yang P. Deep Neural Network Based Artificial Intelligence Assisted Diagnosis of Bone Scintigraphy for Cancer Bone Metastasis. Sci Rep (2020) 10(1):17046. doi: 10.1038/s41598-020-74135-4 33046779 PMC7550561

[93] Cheng DC Hsieh TC Yen KY Kao CH. Lesion-Based Bone Metastasis Detection in Chest Bone Scintigraphy Images of Prostate Cancer Patients Using Pre-Train, Negative Mining, and Deep Learning. Diagn. (Basel) (2021) 11(3):518. doi: 10.3390/diagnostics11030518 PMC8000593 33803921

[94] Arunachalam HB Mishra R Daescu O Cederberg K Rakheja D Sengupta A. Viable and Necrotic Tumor Assessment From Whole Slide Images of Osteosarcoma Using Machine-Learning and Deep-Learning Models. PloS One (2019) 14(4):e0210706. doi: 10.1371/journal.pone.0210706 30995247 PMC6469748

[95] Fu Y Xue P Ji H Cui W Dong E. Deep Model With Siamese Network for Viable and Necrotic Tumor Regions Assessment in Osteosarcoma. Med Phys (2020) 47(10):4895–905. doi: 10.1002/mp.14397 32677073

[96] Nabid RA Rahman ML Hossain MF Ieee "Classification of Osteosarcoma Tumor From Histological Image Using Sequential RCNN", in: 11th International Conference on Electrical and Computer Engineering (ICECE). Dhaka, Bangladesh Publisher: IEEE (2020) pp. 363–6.

[97] Foersch S Eckstein M Wagner DC Gach F Woerl AC Geiger J. Deep Learning for Diagnosis and Survival Prediction in Soft Tissue Sarcoma. Ann Oncol (2021) 32(9):1178–87. doi: 10.1016/j.annonc.2021.06.007 34139273

[98] Vanel D Bittoun J Tardivon A. MRI of Bone Metastases. Eur Radiol (1998) 8(8):1345–51. doi: 10.1007/s003300050549 9853211

[99] Lecouvet FE Larbi A Pasoglou V Omoumi P Tombal B Michoux N. MRI for Response Assessment in Metastatic Bone Disease. Eur Radiol (2013) 23(7):1986–97. doi: 10.1007/s00330-013-2792-3 23455764

[100] Chen B Zeng Y Liu B Lu G Xiang Z Chen J. Risk Factors, Prognostic Factors, and Nomograms for Distant Metastasis in Patients With Newly Diagnosed Osteosarcoma: A Population-Based Study. Front Endocrinol (Lausanne) (2021) 12:672024. doi: 10.3389/fendo.2021.672024 34393996 PMC8362092

[101] El-Hennawy G Moustafa H Omar W Elkinaai N Kamel A Zaki I. Different (18) F-FDG PET Parameters for the Prediction of Histological Response to Neoadjuvant Chemotherapy in Pediatric Ewing Sarcoma Family of Tumors. Pediatr Blood Cancer (2020) 67(11):e28605. doi: 10.1002/pbc.28605 32706520

[102] Saleh MM Abdelrahman TM Madney Y Mohamed G Shokry AM Moustafa AF. Multiparametric MRI With Diffusion-Weighted Imaging in Predicting Response to Chemotherapy in Cases of Osteosarcoma and Ewing's Sarcoma. Br J Radiol (2020) 93(1115):20200257. doi: 10.1259/bjr.20200257 32706980 PMC8519644

[103] Huang B Wang J Sun M Chen X Xu D Li ZP. Feasibility of Multi-Parametric Magnetic Resonance Imaging Combined With Machine Learning in the Assessment of Necrosis of Osteosarcoma After Neoadjuvant Chemotherapy: A Preliminary Study. BMC Cancer (2020) 20(1):322. doi: 10.1186/s12885-020-06825-1 32293344 PMC7161007

[104] Kim J Jeong SY Kim BC Byun BH Lim I Kong CB. Prediction of Neoadjuvant Chemotherapy Response in Osteosarcoma Using Convolutional Neural Network of Tumor Center (18)F-FDG PET Images. Diagn. (Basel) (2021) 11(11):1976. doi: 10.3390/diagnostics11111976 PMC8617812 34829324

[105] Smrke A Tam YB Anderson PM Jones RL Huang PH. The Perplexing Role of Immuno-Oncology Drugs in Osteosarcoma. J Bone Oncol (2021) 31:100400. doi: 10.1016/j.jbo.2021.100400 34786332 PMC8577488

[106] Raskin KA Schwab JH Mankin HJ Springfield DS Hornicek FJ. Giant Cell Tumor of Bone. J Am Acad Orthop. Surg (2013) 21(2):118–26. doi: 10.5435/jaaos-21-02-118 23378375

[107] He Y Guo J Ding X van Ooijen PMA Zhang Y Chen A. Convolutional Neural Network to Predict the Local Recurrence of Giant Cell Tumor of Bone After Curettage Based on Pre-Surgery Magnetic Resonance Images. Eur Radiol (2019) 29(10):5441–51. doi: 10.1007/s00330-019-06082-2 30859281

[108] Liu Y Yang P Pi Y Jiang L Zhong X Cheng J. Automatic Identification of Suspicious Bone Metastatic Lesions in Bone Scintigraphy Using Convolutional Neural Network. BMC Med Imaging (2021) 21(1):131. doi: 10.1186/s12880-021-00662-9 34481459 PMC8417997

[109] Mercolini F Zucchetta P Jehanno N Corradini N Van Rijn RR Rogers T. Role of (18)F-FDG-PET/CT in the Staging of Metastatic Rhabdomyosarcoma: A Report From the European Paediatric Soft Tissue Sarcoma Study Group. Eur J Cancer (2021) 155:155–62. doi: 10.1016/j.ejca.2021.07.006 34385068

[110] Davila D Antoniou A Chaudhry MA. Evaluation of Osseous Metastasis in Bone Scintigraphy. Semin Nucl Med (2015) 45(1):3–15. doi: 10.1053/j.semnuclmed.2014.07.004 25475375

[111] Elfarra FG Calin MA Parasca SV. Computer-Aided Detection of Bone Metastasis in Bone Scintigraphy Images Using Parallelepiped Classification Method. Ann Nucl Med (2019) 33(11):866–74. doi: 10.1007/s12149-019-01399-w 31493203

[112] Kadomoto S Yaegashi H Nakashima K Iijima M Kawaguchi S Nohara T. Quantification of Bone Metastasis of Castration-Resistant Prostate Cancer After Enzalutamide and Abiraterone Acetate Using Bone Scan Index on Bone Scintigraphy. Anticancer Res (2019) 39(5):2553–9. doi: 10.21873/anticanres.13377 31092452

[113] Cheng D-C Liu C-C Hsieh T-C Yen K-Y Kao C-H. Bone Metastasis Detection in the Chest and Pelvis From a Whole-Body Bone Scan Using Deep Learning and a Small Dataset. Electronics (2021) 10(10):1201. doi: 10.3390/electronics10101201

[114] Han S Oh JS Lee JJ. Diagnostic Performance of Deep Learning Models for Detecting Bone Metastasis on Whole-Body Bone Scan in Prostate Cancer. Eur J Nucl Med Mol Imaging. (2021) 49(2):585–95. doi: 10.1007/s00259-021-05481-2 34363089

[115] Masoudi S Mehralivand S Harmon SA Lay N Lindenberg L Mena E. Deep Learning Based Staging of Bone Lesions From Computed Tomography Scans. IEEE Access (2021) 9:87531–42. doi: 10.1109/access.2021.3074051 PMC8562651 34733603

[116] Fan X Zhang X Zhang Z Jiang Y. Deep Learning-Based Identification of Spinal Metastasis in Lung Cancer Using Spectral CT Images. Sci Prog. (2021) 2021:7. doi: 10.1155/2021/2779390

[117] Fan X Zhang X Zhang Z Jiang Y. Deep Learning on MRI Images for Diagnosis of Lung Cancer Spinal Bone Metastasis. Contrast Media Mol Imaging (2021) 2021:5294379. doi: 10.1155/2021/5294379 34354553 PMC8294999

[118] Shang LW Ma DY Fu JJ Lu YF Zhao Y Xu XY. Fluorescence Imaging and Raman Spectroscopy Applied for the Accurate Diagnosis of Breast Cancer With Deep Learning Algorithms. BioMed Opt. Express (2020) 11(7):3673–83. doi: 10.1364/boe.394772 PMC7510916 33014559

[119] Tomczak K Czerwińska P Wiznerowicz M. The Cancer Genome Atlas (TCGA): An Immeasurable Source of Knowledge. Contemp. Oncol (Pozn) (2015) 19(1a):A68–77. doi: 10.5114/wo.2014.47136 PMC4322527 25691825

[120] Network, C.G.A.R. Comprehensive and Integrated Genomic Characterization of Adult Soft Tissue Sarcomas. Cell (2017) 171(4):950–965.e928. doi: 10.1016/j.cell.2017.10.014 29100075 PMC5693358

[121] Deepak S Ameer PM. Brain Tumor Classification Using Deep CNN Features *via* Transfer Learning. Comput Biol Med (2019) 111:103345. doi: 10.1016/j.compbiomed.2019.103345 31279167

[122] Avanzo M Wei L Stancanello J Vallières M Rao A Morin O. Machine and Deep Learning Methods for Radiomics. Med Phys (2020) 47(5):e185–202. doi: 10.1002/mp.13678 PMC8965689 32418336

[123] Ismail FW Shamsudin AM Wan Z Daud SM Samarendra MS. Ki-67 Immuno-Histochemistry Index in Stage III Giant Cell Tumor of the Bone. J Exp Clin Cancer Res (2010) 29(1):25. doi: 10.1186/1756-9966-29-25 20226047 PMC2848627

[124] Zhao J Dean DC Hornicek FJ Yu X Duan Z. Emerging Next-Generation Sequencing-Based Discoveries for Targeted Osteosarcoma Therapy. Cancer Lett (2020) 474:158–67. doi: 10.1016/j.canlet.2020.01.020 31987920

[125] Zeng Z Mao C Vo A Li X Nugent JO Khan SA. Deep Learning for Cancer Type Classification and Driver Gene Identification. BMC Bioinf (2021) 22(Suppl 4):491. doi: 10.1186/s12859-021-04400-4 PMC8543824 34689757

[126] Zeng M Zhou J Wen L Zhu Y Luo Y Wang W. The Relationship Between the Expression of Ki-67 and the Prognosis of Osteosarcoma. BMC Cancer (2021) 21(1):210. doi: 10.1186/s12885-021-07880-y 33648449 PMC7923819

[127] Kong H Yu W Chen Z Li H Ye G Hong J. CCR9 Initiates Epithelial-Mesenchymal Transition by Activating Wnt/beta-Catenin Pathways to Promote Osteosarcoma Metastasis. Cancer Cell Int (2021) 21(1):648. doi: 10.1186/s12935-021-02320-0 34863167 PMC8642956

[128] Akagi M Nakamura Y Higaki T Narita K Honda Y Zhou J. Deep Learning Reconstruction Improves Image Quality of Abdominal Ultra-High-Resolution CT. Eur Radiol (2019) 29(11):6163–71. doi: 10.1007/s00330-019-06170-3 30976831

[129] Kim JH Yoon HJ Lee E Kim I Cha YK Bak SH. Validation of Deep-Learning Image Reconstruction for Low-Dose Chest Computed Tomography Scan: Emphasis on Image Quality and Noise. Korean J Radiol (2021) 22(1):131–8. doi: 10.3348/kjr.2020.0116 PMC7772377 32729277

[130] Coudray N Ocampo PS Sakellaropoulos T Narula N Snuderl M Fenyo D. Classification and Mutation Prediction From non-Small Cell Lung Cancer Histopathology Images Using Deep Learning. Nat Med (2018) 24(10):1559–67. doi: 10.1038/s41591-018-0177-5 PMC9847512 30224757

[131] Yu KH Wang F Berry GJ Re C Altman RB Snyder M. Classifying non-Small Cell Lung Cancer Types and Transcriptomic Subtypes Using Convolutional Neural Networks. J Am Med Inform Assoc (2020) 27(5):757–69. doi: 10.1093/jamia/ocz230 PMC7309263 32364237

[132] Liu S Yao W. Prediction of Lung Cancer Using Gene Expression and Deep Learning With KL Divergence Gene Selection. BMC Bioinf (2022) 23(1):175. doi: 10.1186/s12859-022-04689-9 PMC9103042 35549644

[133] Wang Y Kartasalo K Weitz P Acs B Valkonen M Larsson C. Predicting Molecular Phenotypes From Histopathology Images: A Transcriptome-Wide Expression-Morphology Analysis in Breast Cancer. Cancer Res (2021) 81(19):5115–26. doi: 10.1158/0008-5472.CAN-21-0482 PMC9397635 34341074

[134] Li Z Li Z Chen Q Ramos A Zhang J Boudreaux JP. Detection of Pancreatic Cancer by Convolutional-Neural-Network-Assisted Spontaneous Raman Spectroscopy With Critical Feature Visualization. Neural Netw (2021) 144:455–64. doi: 10.1016/j.neunet.2021.09.006 34583101

[135] Shu C Yan H Zheng W Lin K James A Selvarajan S. Deep Learning-Guided Fiberoptic Raman Spectroscopy Enables Real-Time *In Vivo* Diagnosis and Assessment of Nasopharyngeal Carcinoma and Post-Treatment Efficacy During Endoscopy. Anal Chem (2021) 93(31):10898–906. doi: 10.1021/acs.analchem.1c01559 34319713

[136] Koya SK Brusatori M Yurgelevic S Huang C Werner CW Kast RE. Accurate Identification of Breast Cancer Margins in Microenvironments of Ex-Vivo Basal and Luminal Breast Cancer Tissues Using Raman Spectroscopy. Prostaglandins Other Lipid Mediat. (2020) 151:106475. doi: 10.1016/j.prostaglandins.2020.106475 32711127

[137] Shin H Oh S Hong S Kang M Kang D Ji YG. Early-Stage Lung Cancer Diagnosis by Deep Learning-Based Spectroscopic Analysis of Circulating Exosomes. ACS Nano (2020) 14(5):5435–44. doi: 10.1021/acsnano.9b09119 32286793

[138] Yu M Yan H Xia J Zhu L Zhang T Zhu Z. Deep Convolutional Neural Networks for Tongue Squamous Cell Carcinoma Classification Using Raman Spectroscopy. Photodiagnosis Photodyn Ther (2019) 26:430–5. doi: 10.1016/j.pdpdt.2019.05.008 31082525

